# Nutritional support contributes to improving cardiac function and nutritional status in atrial fibrillation patients receiving treatment with esmolol hydrochloride

**DOI:** 10.3389/fcvm.2025.1721246

**Published:** 2025-12-04

**Authors:** Xiaochao Liu, Yulong Fan, Zhi Liu

**Affiliations:** 1 Department of Emergency, Xuanwu Hospital Capital Medical University, Beijing, China; 2 Nerve Intervention Center, Capital Medical University Affiliated Tongren Hospital Mentougou Hospital, Beijing, China

**Keywords:** esmolol hydrochloride, atrial fibrillation, nutritional support, nursing care, cardiac function, clinical efficacy

## Abstract

**Aim:**

This study focused on the effect of nutritional support combined with esmolol hydrochloride on the efficacy, nutritional status and quality of life of patients with atrial fibrillation (AF).

**Methods:**

This retrospective cohort study categorised patients into a treatment group (TG) and a nursing group (NG) based on whether they received a high-quality care model, spanning the period from February 2021 to February 2024. Both groups received treatment with esmolol hydrochloride. The TG received standard care, while the NG received enhanced care (nutritional intervention + skin care) in addition to standard care. The study compared efficacy, cardiac function, nutritional status, adverse reactions, care satisfaction, and quality of life between the two groups.

**Result:**

Each group included 93 patients. The clinical efficacy of esmolol hydrochloride treatment in AF patients exceeds 90%, with nutritional care showing no significant enhancement of clinical outcomes (*P* = 0.249). However, compared to esmolol monotherapy, combined nutritional care intervention significantly reduced the length of hospitalisation, elevated Left ventricular ejection fraction (LVEF) levels, reduced Left ventricular systolic vector (LVESV) levels, and markedly improved nutritional indicators including Albumin (ALB), Total protein (TP), and Prognostic Nutritional Index (PNI). Furthermore, the combined intervention group demonstrated markedly improved nursing satisfaction and quality of life.

**Conclusion:**

Nutritional care administered alongside treatment with esmolol hydrochloride demonstrates superior efficacy in improving cardiac function, nutritional status, and quality of life among patients with AF.

## 1 Introduction

Atrial fibrillation (AF) and atrial flutter (AFL) are two common clinical arrhythmias, of which atrial fibrillation is the most common (1). Atrial fibrillation presents clinically with palpitations, angina pectoris, paroxysmal dyspnoea or low cerebral output (2). Atrial fibrillation seriously affects the quality of life of patients, and it can cause a series of complications such as stroke, heart failure, cardiac arrest and so on, which increases the economic and psychological burden of patients, and in serious cases, causes death (3). Statistical data indicates that over 33 million people worldwide are affected, with a lifetime risk as high as 25% (4). Current treatments for atrial fibrillation include drug therapy, catheter ablation therapy, and maze procedure. The maze procedure, regarded as the gold standard for surgical treatment of AF, primarily achieves its effect by establishing a series of precise electrical isolation barriers within the atria to block the abnormal electrical pathways responsible for AF. This approach boasts a high success rate and has been shown to reduce morbidity and mortality. However, the procedure is complex and invasive, presenting certain limitations in its application (5). Pharmacological treatments include drugs to restore normal sinus rhythm such as procainamide and quinidine; drugs to control ventricular velocities such as receptor blockers; and drugs to prevent thrombosis and stroke such as antiplatelet anticoagulants. However, all of these drugs have individual variability in adverse effects (6).

Esmolol hydrochloride is a beta-blocker (BB) drug, belonging to the second class of antiarrhythmic drugs, with fast onset of action (2 min) and short half-life (9 min). It is the most commonly used beta-blocker in the treatment of atrial fibrillation. It can effectively treat atrial fibrillation, angina pectoris, tachycardia and hypertension (7, 8). The American College of Cardiology/American Heart Association Task Force recommended the use of BB drugs in the perioperative period to reduce cardiac risk, and esmolol hydrochloride is the way forward for perioperative alternatives to BB drugs due to its potential role in opioid savings (9, 10). Evidence indicates that the long-acting beta-blocker metoprolol dominates BB prescriptions in China (62.2%) (11). Conversely, esmolol, as an ultra-short-acting BB, has been shown in studies to be non-inferior to metoprolol in restoring patients' heart rate and blood pressure, whilst achieving recovery more rapidly (12). This suggests esmolol offers greater advantages for patients requiring precise dose titration or those in unstable conditions (13). Typically, once esmolol demonstrates patient tolerance to BBs and systemic stability, clinicians transition patients to oral BBs for maintenance therapy. Consequently, during the acute phase of improving cardiac function and nutritional status in AF patients, esmolol provides superior safety and controllability.

As AF and AFL are prevalent in the elderly with multiple complications, they are prone to malnutrition and immunodeficiency (14). Nutritional support is important for improving the patient's condition and prognosis, and has become an important adjunct to the treatment of patients with atrial fibrillation (15). Nutritional interventions such as the Mediterranean diet play a crucial part in the recovery and maintenance of health in patients with AF and SFL (16).

As the most commonly used short-acting BB in AF treatment, studies on its application in AF are frequent. We aimed to reduce the adverse reactions of esmolol hydrochloride and improve patients' quality of life. Nutritional intervention has been demonstrated to exert positive effects as an adjunctive measure in AF management. However, its potential to augment the therapeutic efficacy of esmolol hydrochloride remains unexplored. This study therefore incorporates enhanced nursing care (comprising nutritional intervention and skin care) alongside esmolol hydrochloride therapy to investigate the impact of such comprehensive care—particularly nutritional support—on the clinical condition, nutritional status, and quality of life of AF patients receiving esmolol hydrochloride treatment.

## 2 Materials and methods

### 2.1 Study subjects

This retrospective cohort study grouped patients into a Treatment group (TG) and a Nursing group (NG) based on whether they received a high-quality care model. The data for this study were sourced from the electronic medical record system of Xuanwu Hospital, Capital Medical University, covering the period from February 2021 to February 2024. In our hospital, the nutritional intervention programme was introduced as an optional multidisciplinary care pathway in the cardiovascular ward from December 2021. This programme was jointly developed by the Department of Cardiology, Clinical Nutrition Department, and Nursing Department to implement the recommendations for individualised lifestyle interventions outlined in the 2021 ESC Guidelines for the Prevention of Cardiovascular Disease. Patients receiving the nutritional intervention programme were assigned to the NG, whilst those attending prior to this date, those declining the programme, or those with financial constraints were assigned to the TG. TG patients received treatment with esmolol hydrochloride in addition to standard care, whilst NG patients received high-quality care centred on nutritional support alongside esmolol hydrochloride treatment. Inclusion criteria: (1) patients with atrial fibrillation confirmed by electrocardiogram, color Doppler ultrasound and other imaging; (2) no history of relevant drug allergy; (3) confirmed to have received treatment with esmolol hydrochloride, with complete medication records and follow-up data available; (4) age greater than 18 years; (5) Patients with malnutrition scoring less than 45 on the PNI scale. Exclusion criteria: (1) patients with other cardiac diseases, including hypertension and specific cardiac pathologies; (2) patients with functional diseases of liver, kidney and other organs; (3) patients with malignant tumors or other critical illnesses; (4) patients who were unconscious and did not cooperate with the treatment plan. This study received ethical approval from the Ethics Committee of Xuanwu Hospital, Capital Medical University (210017_BX[L]). All participants provided written informed consent to participate in the research. This retrospective cohort study was conducted and reported in accordance with the Standardised Reporting of Observational Studies in Epidemiology (STROBE) statement.

### 2.2 Treatment protocol

Both patients require bed rest and 24 h treatment with esmolol, with comprehensive management of risk factors such as blood pressure and blood glucose levels throughout the process. Intravenous esmolol hydrochloride 0.5 mg/(kg-min) was injected for about 1 min, followed by an intravenous drip maintenance dose of 0.05 mg/(kg-min), and the indexes were observed after 4 min, and the maintenance dose should be increased by 0.05 mg/(kg-min) with a maximum of 0.3 mg/(kg-min) if the efficacy of esmolol was not satisfactory. Vital signs and haemodynamic changes were closely monitored during the esmolol drip.

### 2.3 Nursing methods

TG patients received routine care, including vital signs monitoring, administration of medication as prescribed, dietary guidance, and skin care. Skin care involved regular repositioning of bedridden patients and maintaining clean, dry bedding to prevent pressure ulcers.The NG group was optimised on the basis of conventional care.

#### 2.3.1 Nutritional support

For malnourished patients, oral nutritional supplementation (ONS) needs to be given, with the elemental energy ratio controlled at protein: fat: carbohydrate = 1:2:3, and heat-nitrogen ratio = 130:1. The patient's nutritional status was assessed based on the PNI results.

#### 2.3.2 Nutritional interventions

Nutritional interventions were carried out using the Mediterranean diet. The approach is characterised by the use of only Eva oil in all cooking, high consumption of vegetables, fruits, whole grains, legumes and nuts; moderate consumption of fish; other processed foods and desserts need to be consumed sparingly [1]. The specific protocol is as follows: consume more than 400 g of vegetables and 375 g of fruits per day; consume more than 180 g of legumes, 450 g of fish, and 90 g of nuts per week (recommended to be divided into 3 days' worth); and try to choose whole-grain cereals (like breads, noodles, and rice) as much as possible.

#### 2.3.3 Skin care

Closely monitor the infusion site. Should any signs of phlebitis such as redness, swelling, heat, pain, or tenderness be detected, immediately change the infusion site and apply a warm compress using a 50% magnesium sulphate solution to alleviate symptoms. Additionally, implement appropriate nursing measures during the patient's hospital stay. Nursing measures are as follows: avoid prolonged exposure to sunlight; avoid using soap when bathing and apply moisturising lotion after bathing to avoid drying of the skin; avoid contact with substances that may irritate the skin, such as chemical reagents; when redness, swelling and itching of the skin occurs, try to avoid touching the wound area to cause infection; maintain the temperature of the ward at 23 °C and the humidity at 55%; clean and disinfect the ward daily.

Patients are discharged from the hospital on a continuous basis, following medical advice and continuous care. The patient should return to the hospital on the seventh day and one month after discharge.

### 2.4 Observation indicators

#### 2.4.1 Treatment effect

Observe the therapeutic effect of the two groups, and the effect evaluation is divided into three grades. (a) Obviously effective: clinical symptoms disappear or basically disappear, ectopic rhythm reverts to sinus rhythm, and the ventricular rate of sinus tachycardia slows down to 100 beats/min. (b) Effective: ventricular rate is more than 100 beats/min, but slows down ≥20% compared with the pre-drug period, clinical symptoms are significantly improved, and the ectopic rhythm has not reverted to sinus rhythm. (c) Ineffective: Failure to meet the above criteria or even deterioration (17).

#### 2.4.2 Length of hospitalisation

Compare the length of hospitalisation of the two groups of patients.

#### 2.4.3 Cardiac function indexes

Observe the cardiac function of the two groups of patients before and after drug administration. Including Left ventricular ejection fraction (LVEF), Left ventricular diastolic volume (LVEDV), Left ventricular systolic vector (LVESV) three indicators.

#### 2.4.4 Adverse reactions

Compare the adverse reactions after drug administration in TG and NG groups. These include hypotension, low heart rate, nausea and vomiting, and rash.

#### 2.4.5 Nutritional status

Measure Albumin (ALB), Total protein (TP), Prognostic Nutritional Index (PNI) and Body Mass Index (BMI) in TG and NG groups. Fasting venous blood was collected at the time of admission, 7 days after the nutritional intervention and 1 month after the nutritional intervention for measurement. The nutritional index was calculated as follows: raw score = serum albumin (g/L) + 5 × total lymphocyte count (10^9^/L). PNI ≤ 38 was considered malnutrition, and PNI > 38 was considered normal nutrition (18).

#### 2.4.6 Quality of life assessment

The Short Form 36 Health Survey (SF-36) scale was applied to assess patients' cognitive, psychological, emotional and other functions, with a total score of 100. The score was proportional to the quality of life.

#### 2.4.7 Nursing care satisfaction

A self-made satisfaction questionnaire was used to investigate the nursing care satisfaction of patients in the TG and NG groups. This questionnaire comprises three sections: assessment of nursing staff service attitude, evaluation of nursing quality, and appraisal of health education effectiveness. The total score is out of 100 points, categorised as follows: Very Satisfied (≥90 points) Satisfied (70–90 points) Not Satisfied (<70 points).

### 2.5 Statistical analyses

SPSS 20.0 was used to process and analyse the data, and GraphPad Prism 8.0 was used to plot the graphs. Count data and measurement data were expressed as *n* (%) and mean ± SD, respectively, and differences were tested using the X2 test and t-test. Multivariate linear regression analysis was employed to evaluate the association between nutritional intervention and changes in cardiac function, nutritional status, and quality of life scores, while adjusting for variables such as age, gender, and disease duration. For binary outcomes (treatment response, adverse events), logistic regression analysis was utilized. A two-tailed *P* value < 0.05 was considered statistically significant.

## 3 Results

A total of 186 patients with atrial fibrillation underwent inclusion assessment, with no patient dropouts during follow-up. Among these, 101 were males and 85 were females, with ages ranging from 20 to 69 years old. Comparison of general information such as gender, age and duration of disease among the two groups showed no statistically significant differences (*P* > 0.05, Table 1).

**Table 1:** Demographic characteristics and disease duration of the two groups of patients.

Items	TG (*n* = 93)	NG (*n* = 93)	*P* value
Gender(male/female)	42/51	43/50	>0.05
Average BMI (kg/m^2^)	22.66 ± 2.2	22.71 ± 2.23	>0.05
Average age (years)	63.54 ± 7.54	63.85 ± 7.66	>0.05
Cardiac functional grade			
Grade I	55	57	>0.05
Grade II	29	29	>0.05
Grade III	9	7	>0.05
Average disease course (years)	2.75 ± 0.51	2.76 ± 0.53	>0.05

To control for the potential influence of confounding factors on the estimation of nursing intervention effects, we constructed multivariate regression models with nutritional intervention (NG = 1, TG = 0) as the primary exposure variable, while simultaneously adjusting for the following covariates: age, gender, disease duration, and heart function classification. Depending on the nature of the outcome variables, we employed either multiple linear regression or logistic regression for analysis.

### 3.1 Comparison of clinical efficacy before and after treatment

**Figure 1:**
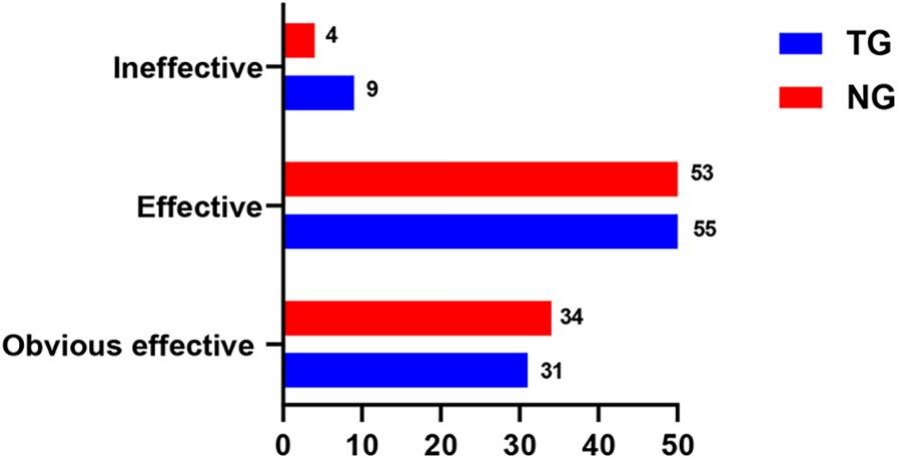
Comparison of clinical efficacy before and after treatment in both groups.

As shown in Figure 1, the clinical efficacy of TG (90.32%) and NG (95.70%) was greater than 90%, and the efficacy of NG had a tendency to increase compared with TG, but there was no statistically significant difference (Adjusted OR = 1.1, 95% CI:0.83–1.75, *P* = 0.249).

### 3.2 Comparison of length of hospitalization

**Figure 2:**
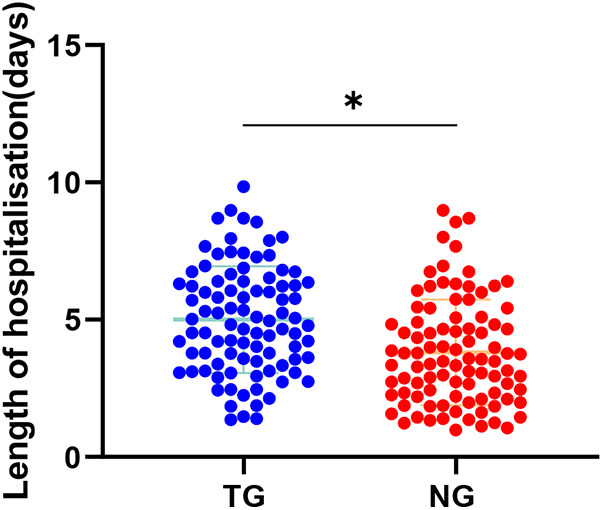
Comparison of length of hospitalization in both groups. *P < 0.05, * represents vs. TG.

As shown in Figure 2, the mean length of hospitalization in TG (mean = 5.02) was significantly higher than the mean length of hospitalization in NG (mean = 3.81) (Adjusted *β* = −0.56, 95% CI:−1.17–0.28, *P* < 0.05).

### 3.3 Comparison of cardiac function before and after treatment

**Figure 3:**
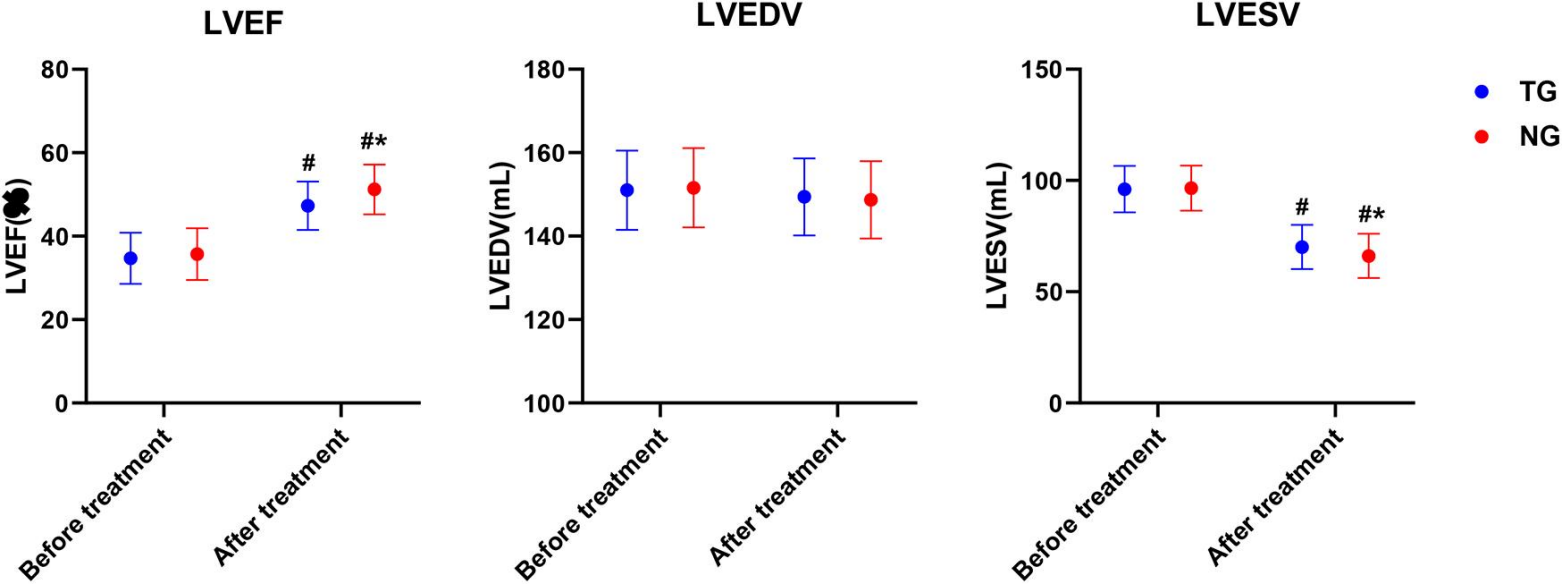
Comparison of cardiac function before and after treatment in both groups. #*P* < 0.05. # represents after treatment vs. before treatment in the same group, *P < 0.05, * represents vs. TG.

As shown in Figure 3, the LVEF index of TG and NG was significantly higher than that before treatment, LVESV were significantly lower than that before treatment, and the LVEF (Adjusted *β* = 0.78, 95% CI:0.52–1.2, *P* < 0.05).and LVESV (Adjusted *β* = −0.51, 95% CI:−0.98–0.37, *P* < 0.05).of NG was better than that of TG.

### 3.4 Comparison of adverse reactions before and after nursing

Patients may experience adverse reactions such as hypotension, low heart rhythm, nausea and vomiting, and rash during the use of esmolol hydrochloride. The incidence of adverse reactions in the NG was notably lower than that in the TG (Adjusted OR = 0.45, 95% CI:0.24–0.95, *P*  =  0.027), as shown in Table 2.

**Table 2:** Comparison of adverse reactions before and after nursing between the two groups.

Groups	Hypotension	Bradycardia	Nausea and vomiting	Pruritus	Total incidence rates
TG (*n* = 93)	6 (6.45)	5 (5.38)	3 (3.23)	7 (7.53)	21 (22.58)
NG (*n* = 93)	4 (4.30)	4 (4.30)	1 (1.07)	0 (0)	9 (9.68)
*χ*2	–	–	–	–	–
*P*	0.747	1	0.621	0.014	0.027

### 3.5 Comparison of nutritional status before and after nursing

**Figure 4:**
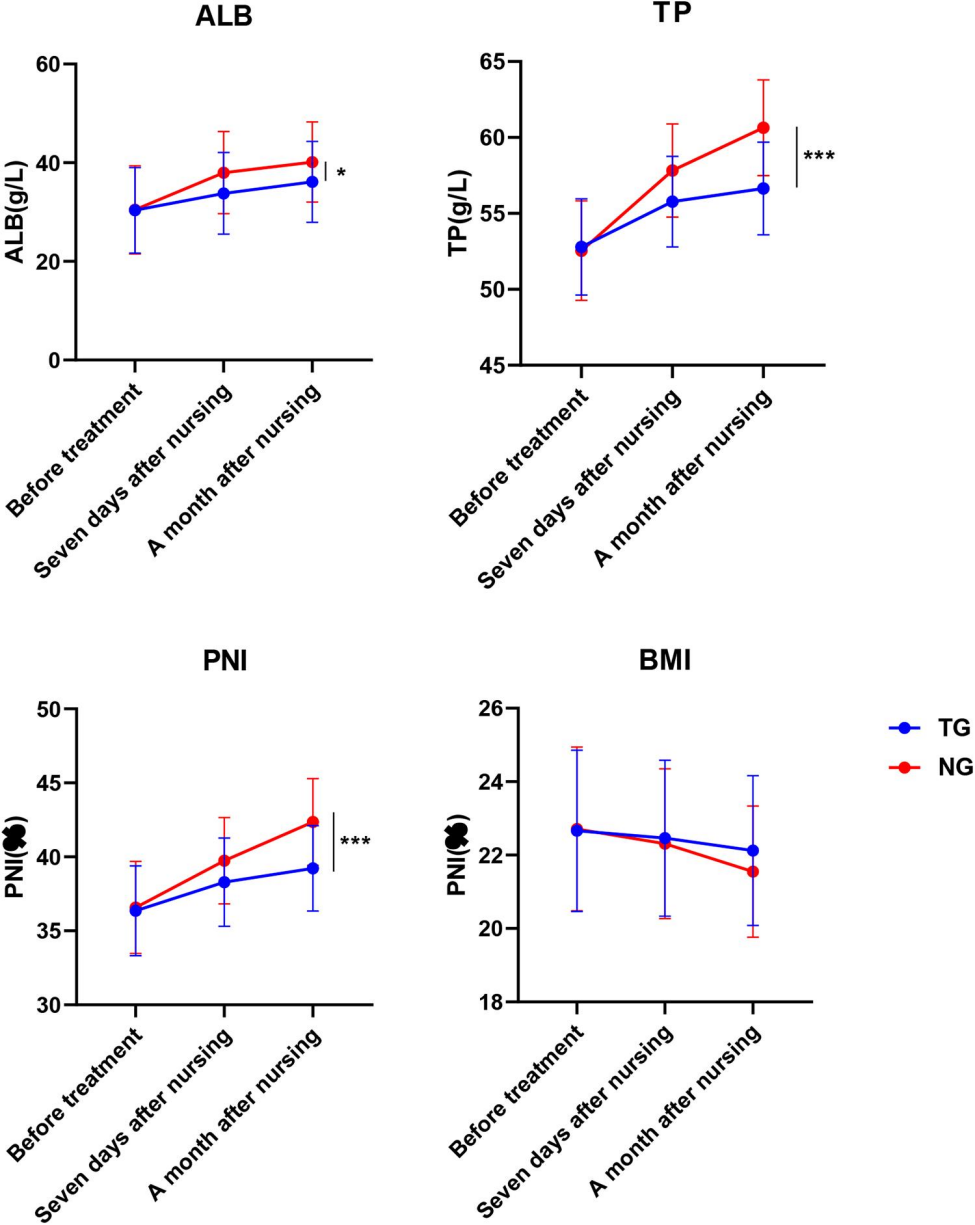
Comparison of nutritional status before and after nursing in both groups. *P < 0.05, ***P < 0.001, * represents vs. TG.

The nutritional status of the patients before admission, after one week of nutritional intervention and after one month of nutritional intervention were detected respectively. The ALB (Adjusted *β* = 3.2, 95% CI:1.5–5.1, *P* < 0.05), TP (Adjusted *β* = 5.3, 95% CI:2.9–7.7, *P* < 0.001)and PNI (Adjusted *β* = 6.9, 95% CI:4.3–9.7, *P* < 0.01) of the NG were notably higher than that of the TG. Nutritional levels after one month of care showed an increasing trend compared to those after one week of care. The BMI of patients in the NG group was significantly lower after care than before care (*P* < 0.05), and there was a decreasing trend compared to TG, but it was not statistically significant (Adjusted *β* = −0.12, 95% CI:−0.52–0.22, *P* > 0.05), as shown in Figure 4.

### 3.6 Comparison of satisfaction with care before and after nursing

**Figure 5:**
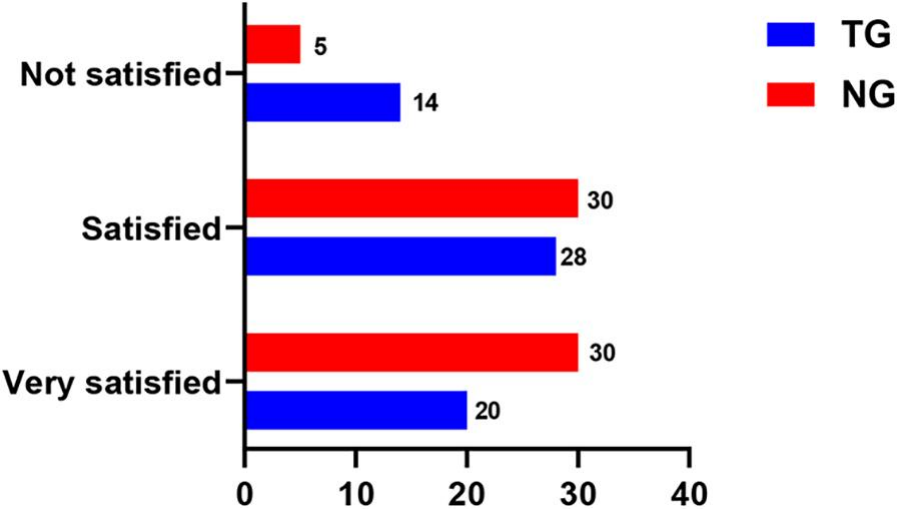
Comparison of satisfaction before and after nursing in both groups.

Nursing care satisfaction was notably higher in the NG than in the TG (Adjusted *β* = 5.7, 95% CI:3.2–9.4, *P* < 0.001)), as shown in Figure 5.

### 3.7 Assessment of quality of life

**Figure 6:**
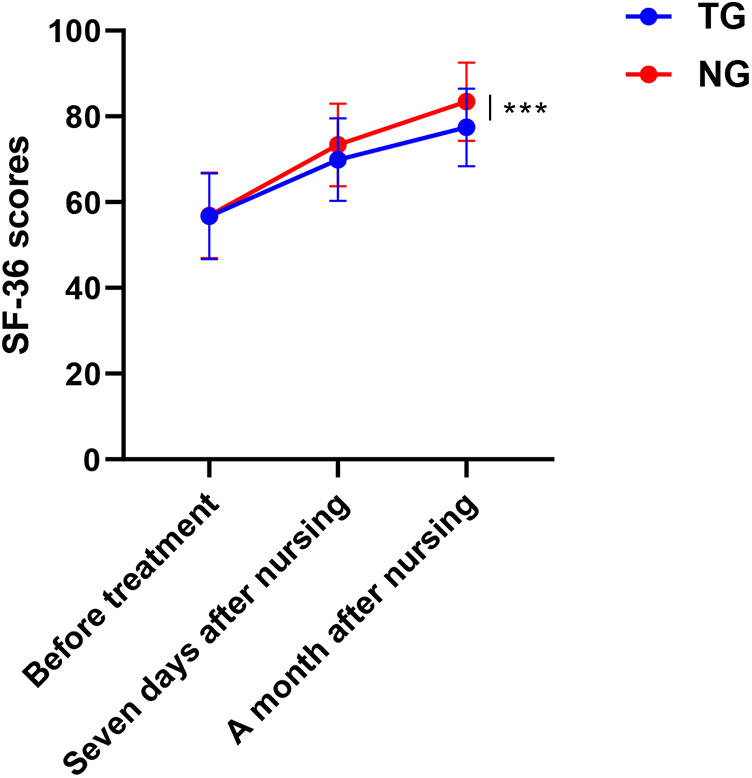
Comparison of quality of life in both groups. ***P < 0.001, * represents vs. TG.

The quality of life in the NG was notably higher than that in the TG (Adjusted *β* = 9.2, 95% CI:6.9–13.4, *P* < 0.001), as shown in Figure 6.

## 4 Discussion

Numerous risk factors contribute to atrial fibrillation. Unfavourable lifestyle choices and prolonged hospital stays may shorten cardiac filling time, thereby impairing left ventricular function and increasing the likelihood of heart failure and poor haemodynamic performance (19). As one of the commonly used therapeutic agents, beta-blockers primarily exert their effects through competitive inhibition of beta-adrenergic receptors (*β*-ar), producing negative inotropic effects, reducing myocardial oxygen consumption, and preventing cardiac damage from excessive sympathetic activation, thereby improving ventricular and vascular remodelling (20). Aceloridol hydrochloride is the most frequently employed short-acting BB for treating AF, with multiple studies confirming its efficacy in managing and ameliorating the condition (21, 22). Among the 186 retrospective cases in this study, treatment with aceloridol hydrochloride demonstrated a therapeutic efficacy rate of 90% in AF patients, further substantiating its effectiveness.

In general, a rapid ventricular rate can further induce heart failure in tachycardia cardiomyopathy, and heart failure is the most common complication of AF (23). Heart failure patients suffer from malnutrition due to insufficient nutritional intake, macromolecular metabolism of the body and pancreatic islet resistance. Malnutrition leads to low body resistance which induces multiple complications and ultimately causes death (24, 25). Therefore, nutritional intervention in patients with AF is necessary. In this study, enteral nutrition was given to malnourished patients, and Mediterranean diet was given to normal nutrition patients. The results showed that nutritional intervention was effective in improving the levels of ALB, TP and PIN, as well as BMI in patients with AF (*P* < 0.05). In addition, the longer the duration of nutritional intervention, the more nutritional levels and BMI normalised. Therefore, overweight BMI increases the risk of atrial fibrillation and necessitates nutritional care for patients with AF.

Moreover, we observed that nutritional support significantly shortened hospital stays and improved cardiac function. Evidence indicates that nutritional status constitutes a critical prognostic factor in patients with heart failure, with those exhibiting abnormal LVEF demonstrating a markedly increased likelihood of compromised nutritional status (26). In a controlled clinical trial, Mediterranean diet intervention for heart failure patients markedly increased both LVEF and quality of life (27). Among patients with valvular atrial fibrillation, low levels of PNI elevate disease risk (28). The aforementioned studies collectively demonstrate the detrimental impact of nutritional abnormalities on cardiac function in cardiovascular disease. Research examining the effects of nutritional interventions on cardiac function-related indicators in AF remains limited. This study indicates that nutritional intervention can enhance LVEF and reduce LVESV in AF patients, underscoring the importance of nutritional support for cardiac function in this population. Furthermore, providing nutritional support to individuals at risk of malnutrition significantly improves clinical outcomes such as survival rates (29). Among treated ST-segment elevation myocardial infarction patients, those with low geriatric nutritional risk scores exhibited longer hospital stays and higher mortality risks (30). These findings collectively demonstrate that nutritional assessment and intervention can markedly improve hospitalisation duration and patient prognosis, consistent with our study outcomes.Due to the therapeutic effect of beta-blockers on skin disorders such as haemangiomas, melanomas and wound healing (31), he can also cause some adverse reactions in the skin. Psoriasis, lichen planus-like eruption (LDE), contact dermatitis, and anaphylactic reactions are common dermatological adverse reactions (32). Adverse effects of esmolol hydrochloride mainly include bradycardia, hypotension, and digestive discomfort (33, 34). Although there are no reports directly mentioning the adverse effects of esmolol in the skin, based on the adverse effects of beta-blockers in the skin. In this study, we have studied hypotension, bradycardia, nausea and vomiting and skin rash as adverse reactions. The results indicates that nursing care may have an ameliorating effect on the adverse effects of esmolol on the skin (*P* < 0.05). The quality of life of the NG was notably better than that of the TG, and the quality-of-life score of the TG was notably higher than that of the CG group (*P* < 0.05). It indicates that esmolol hydrochloride can effectively treat atrial fibrillation and improve patients' quality of life. And nursing care on the basis of treatment can further improve the cardiac function and nutritional level, relieve patients' anxiety and thus promote patients' recovery progress. In conclusion, nutritional care on the basis of drug therapy can further improve the therapeutic efficacy of patients, and has a positive role in promoting the physical and mental health of patients and the disease process (35). Esmolol represents modern medicine's capacity for highly effective intervention during the acute phase of AF, whilst nutritional intervention serves as the cornerstone of long-term management and a protective shield, reducing AF triggers and disease burden. This facilitates a shift in AF treatment from a singular biomedical model towards a more comprehensive bio-psycho-social approach, thereby enhancing the integrated prevention and control of AF and improving patients' quality of life. However, high-quality evidence regarding the impact of nutritional interventions on AF prognosis remains scarce. Future prospective studies are required to validate the efficacy and safety of nutritional intervention strategies.

## 5 Limitations

Firstly, the nursing satisfaction questionnaire employed in this study was independently developed, and as primary data, it remains inaccessible for this research. As an unvalidated instrument, this questionnaire may exhibit unknown reliability and validity, potentially compromising the credibility of the findings. The reliability of this questionnaire also requires further validation. Secondly, the follow-up period in this study was limited to one month, insufficient for assessing long-term nutritional and clinical impacts. Consequently, the generalisability of these conclusions is somewhat constrained. Finally, although we controlled for multiple potential confounding factors through multivariate regression, we cannot entirely rule out the influence of unmeasured confounders (such as timing and healthcare provider proficiency) on the results.

## 6 Conclusion

Studies have shown that nutritional support combined with esmolol hydrochloride can effectively improve the outcome and cardiac function of patients with atrial fibrillation, reduce the length of hospitalisation, decrease the adverse effects of medication, and improve life satisfaction.

## Data Availability

The original contributions presented in the study are included in the article/Supplementary Material, further inquiries can be directed to the corresponding author.
